# Long Spiky Au-Ag Nanostar Based Fiber Probe for Surface Enhanced Raman Spectroscopy

**DOI:** 10.3390/ma15041498

**Published:** 2022-02-17

**Authors:** Guangyuan He, Xiaoyu Han, Shiyi Cao, Kaimin Cui, Qihang Tian, Jihong Zhang

**Affiliations:** 1 State Key Laboratory of Silicate Materials for Architectures, Wuhan University of Technology, 122 Luoshi Road, Wuhan 430070, China; heguangyuan@whut.edu.cn (G.H.); hxy0613@whut.edu.cn (X.H.); siyi@whut.edu.cn (S.C.); kaimin@whut.edu.cn (K.C.); tqh@whut.edu.cn (Q.T.); 2 International School of Materials Science and Engineering, Wuhan University of Technology, 122 Luoshi Road, Wuhan 430070, China; 3 School of Materials Science and Engineering, Wuhan University of Technology, 122 Luoshi Road, Wuhan 430070, China

**Keywords:** noble metal, nanostructure, glass fiber, local surface plasmon resonance

## Abstract

The detection performances of noble metal-based surface enhanced Raman spectroscopy (SERS) devices are determined by the compositions and geometries of the metal nanostructures, as well as the substrates. In the current study, long spiky Au-Ag alloy nanostars were synthesized, and both core diameters and spike lengths were controlled by Lauryl sulfobetaine concentrations (as the nanostructure growth skeleton). The long spiky star geometries were confirmed by transmission electron micrograph images. Elements energy dispersive spectrometer mapping confirmed that Au and Ag elements were inhomogeneously distributed in the nanostructures and demonstrated a higher Ag content at surface for potential better SERS performance. Selected synthesized spiky nanostars were uniformly assembled on multi-mode silica fiber for probe fabrication by silanization. The SERS performance were characterized using crystal violet (CV) and rhodamine 6G (R6G) as analyte molecules. The lowest detection limit could reach as low as 10^−8^ M, with a 6.23 × 10^6^ enhancement factor, and the relationship between analyte concentrations and Raman intensities was linear for both CV and R6G, which indicated the potential qualitative and quantitative molecule detection applications. Moreover, the fiber probes also showed good reproducibility and stability in the ambient atmosphere.

## 1. Introduction

Noble metal (such as silver, gold, platinum, copper, etc.) nanostructures have attracted much attention for their optical and electrical properties (originating from their local surface plasmon resonance (LSPR)) for various applications in recent years [1,2,3]. Surface enhanced Raman scattering (SERS) is one of these important applications for ultra-low or single molecular substance detection [4]. Raman scattering signal from analyte molecules can be exponentially amplified from the electric field resonance between nanostructure surface and attached analyte molecules [5,6] under laser excitation [7]. The SERS analytical method has been studied and applied for trace organic molecules detection, pollutant heavy metal ions monitoring [8], food safety [9], bio-sensing [10,11], and others.

The SERS performances are highly dependent on the components, morphologies, geometries of noble metal nanostructures, and supporting materials [12,13]. In general, special nanostructure geometries provide denser hot spots [14] and larger surface area for analyte attachment for better SERS detection behavior than regular sphere nanoparticles. Moreover, sharp tips in special structures generate a much stronger LSPR electric field than sphere nanoparticles [15], due to size and space effect, for further SERS improvement. Many kinds of nanostructures, such as nanocubes [16], nanorods [17], nanostars [18], octahedral nanoparticles [15], and nanocages [19] have been synthesized and their SERS behavior on planar supporting substrates have been investigated [20]. The results indicated that these nanostructures could have higher enhancement factors and reduce the detection limit to 10^−^^9^ M scale for ultra-trace substance analysis [21,22]. In addition, the nanostructure components also are of great importance for SERS performance [23]. Pure Ag nanostructures showed better LSPR effect and SERS detection limit due to higher free electron density and broader UV-visible response range [24]. However, Ag nanostructures are easily oxidized, and the stability of Ag nanostructure-based SERS detection is still a critical problem to be solved [25]. The Raman signal rapidly decayed and disappeared in a short duration (such as 24 h in an ambient atmosphere). Au nanostructures show better stability while their LSPR effect and SERS detection performance were not as good as that from Ag under similar sizes or structures [26]. Au-Ag alloy nanostructures provide both a good LSPR effect and good stabilities, for potential SERS substrates, for ultra-trace molecules detection under different circumstances [27,28,29]. In addition, the spiky stars also could furtherly improve detection limit, from high LSPR induced field strength on sharp tips and large surface area for analyte attachment [30,31].

Silica glass fiber has been extensively applied for information communication mediums due to low optical loss, compact dimensions, lightweight, anti-electromagnetic interference, and environmental stability [32]. In fact, optical fiber can be used as metal nanostructures supporting materials for ultra-trace molecules SERS detection probes [23,33]. The fiber offers input and output optical channel for SERS excitation and detection, more importantly, provides special benefits including flexible, real-time, in-situ, and online spectral detection [34]. Since the excitation beam and SERS signal are confined into the fiber core region, there is a large proportion of scattering signal loss, which result in the detection limit and sensitivity of fiber probes are not as good as planar SERS detector [35]. Recent research found that the detection limit could reach as low as 10^−^^9^ M for silver rod-based fiber probes using 4-aminothiophenol (4-ATP) as analyte [36,37,38] or 10^−^^10^ M for Au-Ag alloy nanostars fiber probes using crystal violet (CV) as analyte [39,40]. Fiber structure designs, such as hollow fiber and tapered fiber, and active nanostructure optimization are potential methods for fiber probe detection improvement [23].

In the present research, long spiky Au-Ag nanostars were synthesized using the metal ions reduction method. The nanostructure geometries, and spiky branch length in particular, were controlled by Lauryl sulfobetaine (LSB) content addition. The sizes, morphologies, and element distributions of nanostructures were analyzed. The obtained long spiky nanostructures were homogeneously coated on the end tip and wall of silica fiber for probes fabrication. The SERS performance was characterized using CV and Rhodamine 6 G as analytes. The reproducibility and stability of fiber probes were investigated as well. The results indicated that long spiky Au-Ag nanostars fiber probes could be applied for low concentration substance analysis.

## 2. Materials and Methods

### 2.1. Materials

Hydrogen tetrachlorocuprate (III) hydrate (HAuCl_4_·3H_2_O, 99.9%), silver nitrate (AgNO_3_, 99.99%), Sodium citrate (C_6_H_5_O_7_Na_3_, 98%), L-dopa (C_9_H_11_NO_4_, 99%), ascorbic acid (C_6_H_8_O_6_, AA, 99%), Lauryl sulfobetaine (C_17_H_37_NO_3_S, LSB, 99%), were purchased from Aladdin (Shanghai, China) and directly used for nanostructure synthesis without further purification. Triethoxy-3-aminopropylsilane (C_9_H_23_NO_3_Si, 3-APTES, 99%, Aladdin) was used as a surfactant for fiber probes fabrication. Dye compounds, rhodamine 6G (C_28_H_31_N_2_O_3_C, 99%, Aladdin), crystal violet (CV) (C_25_H_30_N_3_Cl, 99%, Aladdin) were used as analytes for SERS performance characterization. Deionized (DI) water with a resistivity of 18.2 MΩ·cm produced using a Milli-Q SP ultrapure-water purification system from Nihon Millipore Ltd., Tokyo, Japan was used as a solvent for nanostructure synthesis. Multimode silica optical fibers with 62.5 μm core diameter and 125 μm total diameter were purchased from Yangtze Optical Fiber & Cable Joint Stock Co. Ltd., Wuhan, China.

### 2.2. Synthesis Methods

Long spiky Au-Ag alloy nanostars were synthesized according to Dickson Joseph’s method [41]. 25 mM, 50 mM, 75 mM and 100 mM LSB solutions were prepared by dissolving 0.419, 0.839, 1.258 and 1.678 g LSB, respectively, in 50 mL deionized water and stirred until completely dissolved. 0.5 mL 10 mM HAuCl_4_ solution and 0.05 mL AgNO_3_ (20 mM) solution were added into LSB solution and well mixed by manual stirring. Then, 0.1 mL AA (100 mM), and 0.05 M NaOH solution were added, while continuous magnetic stirring, until the solution turned dark. The solutions were kept at room temperature for 24 h for the growth of Au-Ag alloy nanostars. The solutions were centrifuged and washed using dehydrate ethanol and pure water several times to remove organic and inorganic intermediates, and then concentrated to 2 mL solution, for further use. Since the contents of HAuCl_4_ and AgNO_3_, as raw materials for Au and Ag alloy nanostructures, were fixed, the diameters and branch lengths were determined by LSB concentrations.

Multi-mode silica optical fibers with 62.5 core diameter and 125 cladding thickness were used for fiber probes fabrication. The fibers were soaked into 5 μL/mL 3-APTES aqueous solution for 30 min for surface silanization, then transferred into prepared Au-Ag alloy nanostructures solution for 24 h for long spiky Au-Ag alloy nanostars adsorption in order to fabricate fiber probes (Figure S1). Then, the fabricated fiber probes were dried in vacuum at room temperature.

### 2.3. Materials Characterization

The sizes distribution and morphologies of synthesized metal nanostructures were characterized using transmission electron microscopy (TEM, JEM-1400, JEOL, Japan), with an acceleration voltage of 200 kV. The absorption spectra of the nanostructures were measured using a UV-Vis-NIR spectrophotometer (Lambda 750S, Perkin Elmer, Waltham, MA, USA), ranging from 300 nm to 900 nm. The crystal phase of obtained nanostructures was characterized using an X-Ray diffraction diffractometer (XRD, D8 DISCOVER, Bruker, Germany), operating at 40 kV, 40 mA, with Cu K_α_ radiation, 1.54056 Å, 2°/min scanning speed, and 0.02° step size. The high-resolution TEM images, element distributions mapping, and selected area electron diffraction were taken from another TEM (JEM-2100F, JEOL, Japan), and attached energy dispersive spectroscopy (EDS). The nanostructure distributions on fiber tip were characterized using a field-emission scanning electron microscope (FE-SEM, S-4800, Hitachi, Japan), images were taken using a microscope with a 5 kV accelerating voltage. The digital images of naked fiber and coated fiber were taken using an optical microscope (CX33, Olympus, Japan). All of the measurements were conducted at room temperature.

### 2.4. Electromagnetic Filed Distribution Simulation

The electromagnetic filed distributions around nanostructures were simulated using finite-difference time-domain (FDTD) analysis. The 3D models of the nanostructures were constructed following the TEM images, with individual nanostar, colliding tip-top and intercrossed naonstars, as shown in Figure S2. The excitation wavelength was 633 nm, as the Raman measurement. The air suspension geometrical model n_0_ = 1. The mesh range resolution was set as 1 nm × 1 nm × 1 nm, the monitor position was set as *z*-axis plane, the range was set as 1 μm × 1 μm, the incident light wave vector K was along *z*-axis, the electric field E was along *X*-axis, and E0 = 1 V/m.

### 2.5. SERS Measurements

The obtained fiber probes were dipped into CV or R6G solutions with different concentrations (10^−3^, 10^−4^, 10^−5^, 10^−6^, 10^−7^, 10^−8^, and 10^−9^ M, respectively) for 5 h for further Raman measurement. The SERS performance was characterized by Raman scattering spectra (LABHRev-UV, Horiba, Japan), under 633 nm laser excitation. The laser power was 10.6 mW. The accumulation and integration durations were 3 s and 4 s, respectively. The laser beam was focused on the fiber core center surface through a 50× objective lens, and the beam size was approximately 2 μm. The laser beam propagated through fiber, reached the nanostructure coated end-tip, interacted with analyte molecules and Au-Ag nanostructures. The scattering signal transmitted backward within the fiber and received by the detector follows the setup diagram shown in Figure S3.

## 3. Results

### 3.1. Long Spiky Au-Ag Nanostars

**Figure 1:**
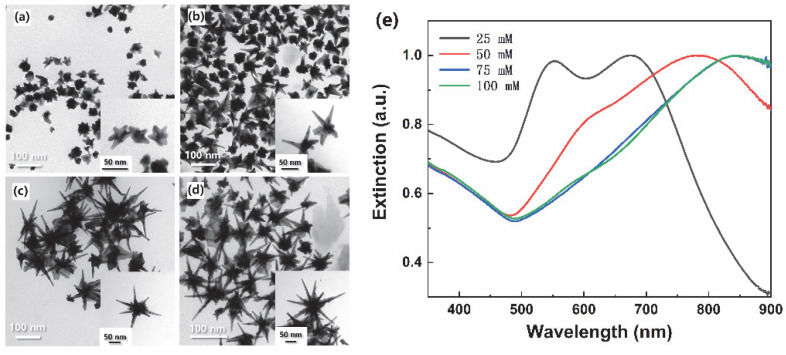
TEM images of nanostructures synthesized with different LSB concentrations: 25 mM (**a**), 50 mM (**b**), 75 mM (**c**), and 100 mM (**d**), inset images were detailed nanostructures geometries of independent particles, and (**e**) extinction spectra of the nanostructures.

The TEM images of synthesized nanostructure morphologies (Figure 1a–d) indicated that spiky tips generated and grew with LSB concentrations increase. Irregular nanoparticles with ~30 nm core diameter and short blunt tips were obtained when LSB concentration was 25 mM (Figure 1a). Spiky tips and larger core diameter (~40 nm) nanostructures were obtained when LSB concentration was 50 mM (Figure 1b). Larger core diameters and longer spiky tips nanostructures were obtained with further LSB concentration increase (Figure 1c,d). Linear LSB molecular structure includes hydrophilic group at one end, and hydrophobic group at another end, which can be assembled into spherical micelles acted as soft templated for spiky nanostructure formation and growth [42]. Considering small particles may provide denser LSPR hot-spot and the difficulty of fiber assembling, spiky nanostructures synthesized with 50 mM LSB were chosen for further fiber probe fabrication and SERS performance investigation. The UV-Vis-NIR spectra (Figure 1e) were consistent with the morphologies of obtained nanostructures. The nanostar synthesized using 25 mM LSB had two main peaks. The peak centered at 550 nm mainly came from the sphere-like core, which was close to the spherical FDTD simulated extinction peak [43] and plasmonic band of spherical gold nanoparticles [44]. The peak centered at 675 nm was derived from the short blunt tips plasmonic band. In fact, the peaks red-shifted with LSB concentration increase, and the sphere-like plasmonic peaks weakened and disappeared, which indicated the formation of long spiky brunches on the larger core. In addition, the extinction peaks from the spiky nanostars synthesized using 50, 75, and 100 mM LSB were similar with those simulated and experimental extinction peaks [43,45] due to similar sizes and geometries. The strong infrared absorption originated from multiple plasmon modes or the longitude propagations mode along with tips, similar to gold nanorod infrared absorption [46]. Moreover, broad peaks indicated the irregular geometries of synthesized nanostructures. The strong infrared absorption also indicated potential infrared SERS applications.

**Figure 2:**
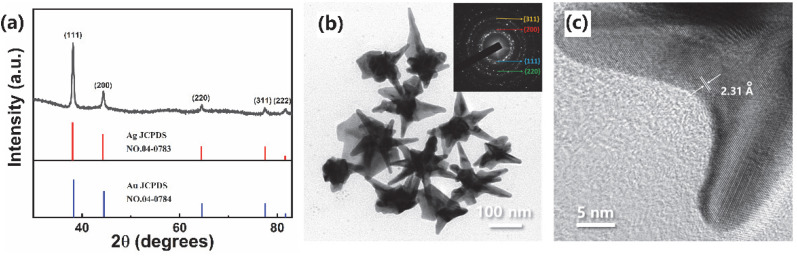
The XRD pattern (**a**), TEM image (**b**), inset was Fourier electron diffraction image, HR-TEM image (**c**), and Au-Ag nanostars synthesized with 50 mM LSB.

**Figure 3:**
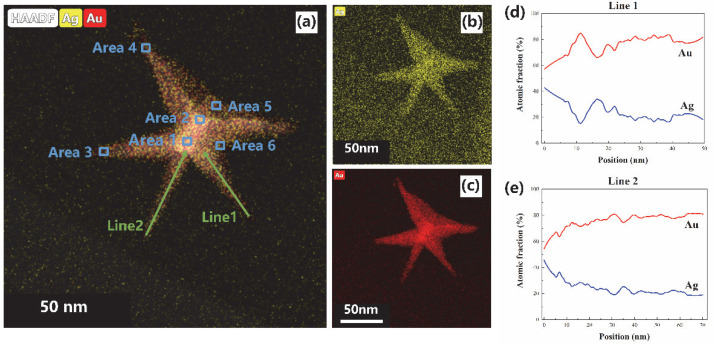
Au and Ag elements distributions of long spiky nanostar (**a**), separated Au (**b**) and Ag (**c**) distribution, and line scanning distribution of the brunch tips line 1 (**d**) and line 2 (**e**). The lines were smoothed.

To confirm the components and element distributions of the obtained nanostructures, the crystal phase structure was investigated by XRD pattern (Figure 2a), high-resolution TEM (Figure 2b,c), EDS analysis (Figure S4), and selected area mapping (Figure 3). The diffraction peaks were assigned to the (1 1 1), (2 0 0), (2 2 0), (3 1 1), and (2 2 2) crystal planes of the face-centered cubic Au [JCPDS 04-0784] and Ag [JCPDS 04-0783] with nearly the same standard patterns. The selected area electron diffraction (SAED) image (Figure 2b inset) also indicated the crystal planes of gold and silver. In addition, the facet had lattice fringes with interplanar space of 0.231 nm, corresponding to the (1 1 1) planes of a faced-centered cubic Au-Ag alloy structure [47,48]. These confirmed the nanostructure constitution of gold and silver. Moreover, the EDS spectrum furtherly confirmed the compositions of silver and gold in nanostructures (Figure S4), and Au-Ag alloy long spiky nanostructure (Figure 3a). The Au and Ag distribution was furtherly analyzed using EDS line scan and area mapping. The whole spiky nanostructure mapping furtherly confirmed the components of Au and Ag (Figure 3a–c). In addition, the line scanning from top spiky branch to bottom showed different Au/Ag molar ratios. Ag concentrations were gradually decreased from brunch top to bottom, while Au increased for both lines 1, and 2 (Figure 3a,d,e). Moreover, the area scanning results showed different Au-Ag concentration distributions at different nanostructure positions (Figure 3a, Table S1). The Au/Ag elements distributions were determined by the nanostructure formation and growth process, which from the inhibiting effect of reduced Ag on Au nanoparticles. In detail, Au^3+^ ions were firstly reduced by ascorbic acid, and Au nanoparticles were formed due to higher reduction potential than that of Ag^+^ ions [48]. Then, reduced Ag clusters were gradually formed and attached to Au nanoparticles, which impeded the further growth of Au nanoparticles. The Au nanoparticles could furtherly grow from the active sites, resulting in the formation of multi-branched star shape and inhomogeneous element distributions. Moreover, long spiky nanostars formation was due to linear LSB molecular as nanostructure growth skeleton. Higher Ag content at spiky brunch top potentially resulted in stronger LSPR electric field intensity and better SERS performance.

**Figure 4:**
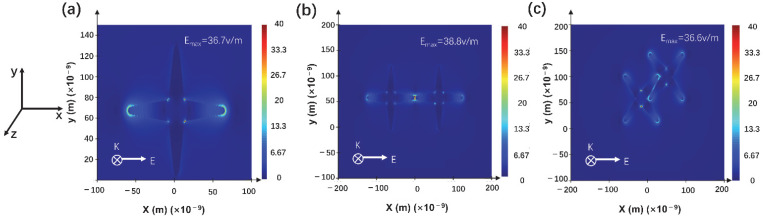
Simulated electric distribution from single nanostar (**a**) colliding tip-top nanostars (**b**) and intercrossed (**c**) long spiky Au-Ag nanostars.

The electromagnetic field distribution of synthesized long spiky nanostars under 633 nm laser excitation was simulated using finite difference time domain (FDTD) analysis. The 3D model was constructed following the TEM image (Figure 1b), with a 40 nm core diameter and 100 nm spiky brunch length, and individual, colliding tip-top, top, and intersecting forms (Figure S2). The laser-induced electric field was along with the *x*-axis. The results indicated that the strongest electric field was at the closed corner of the nanostars due to the nano-gap effect [49] In addition, an intense electric field also was found at the top-tip of the brunch (Figure 4a). For colliding tip-top nanostars, the maximum electric intensity could reach 38.8 V/m, at closed brunch tips gap and nanostar corners (Figure 4b). Moreover, a strong electric field was found at the space of the vertex angles of the two nanostars (Figure 4c), as well as the nanostar corners. The strong electric field intensity and larger distribution area indicated intense and more LSPR hot-spot for better SERS performance.

### 3.2. SERS Performance of Fiber Probes

**Figure 5:**
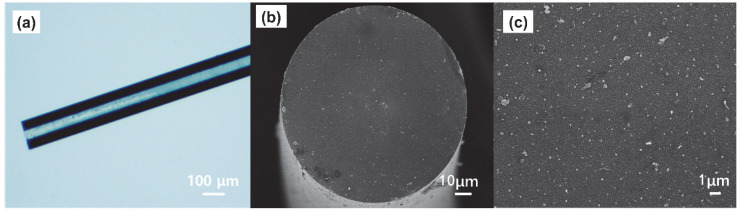
The digital image of SERS fiber probe (**a**), and SEM image of SERS probe with Au-Ag alloy. Nanostars on the terminal end (**b**). The amplification image (**c**).

**Figure 6:**
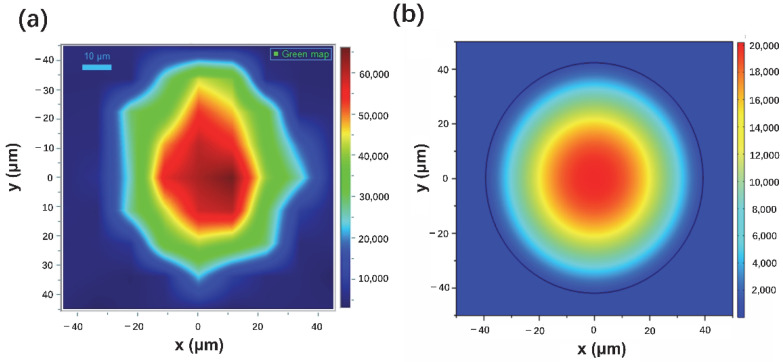
Raman intensities distribution mapping of SERS fiber probe terminal end (**a**) and Raman intensities distribution mapping of blank fiber terminal end (**b**).

The optical microscope images of uncoated fiber showed transparency (Figure S5a) and smooth terminal end surface (Figure S5b), while being dark for Au-Ag spiky nanostructures coated fiber (Figure 5a) from the scattering and absorption from nanostructures, which indicated the presence of nanostructures on the fiber walls. Furthermore, SEM images showed the nanostructures homogeneously and densely coated on the fiber end facet (Figure 5b,c) by surface silanization and electrostatic adsorption. The Raman peaks at 1619 cm^−1^ intensities distributions using 10^−4^ M CV as analyte on the fiber end facet showed a Gaussian-like change from the center to the edge in the fiber core (Figure 6a), which was similar with the intensities from blank fiber using 0.1 M CV as analyte (Figure 6b). Therefore, fiber core centers were chosen for following Raman performance characterization as focused incident beam input positions.

**Figure 7:**
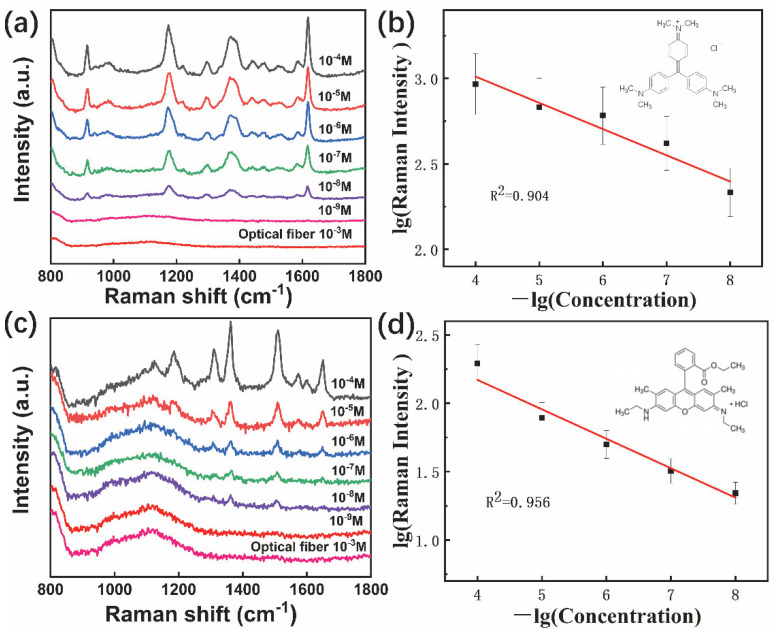
Raman spectra of CV molecules with concentrations from 10^−4^ M to 10^−9^ M, using SERS fiber probes, under 633 nm laser excitation (**a**), and the relationship between CV concentrations and 1619 cm^−1^ peak intensities (**b**), Raman spectra of R6G molecules with concentration from 10^−4^ to 10^−9^ M (**c**), and the relationship between R6G concentrations and 1361 peak intensities (**d**). The optical fiber in (**a**,**c**) indicated Raman spectrum of 10^−3^ M CV or R6G using naked fiber.

The SERS behaviors of fabricated fiber probes were characterized using CV and R6G as analyte molecules, with different concentrations ranging from 10^−4^ to 10^−9^ M. The Raman spectra of CV (Figure 7a) showed that the five main peaks centered at 920, 1171, 1305, 1390, and 1619 cm^−1^ could be assigned to out-of-plane vibration of ring C-H, ring skeletal vibration of radical orientation, in-plane vibration of ring C-H, N-phenyl stretching vibration, and ring C-C stretching vibration, respectively, from the CV molecules [50]. The Raman intensities decreased with a decrease in the concentration until the CV concentration was 10^−9^ M. The lowest detectable concentration or detection limit could be as low as 10^−8^ M for long spiky Au-Ag nanostar-based fiber probe, following the signal and noise ratio larger than three standards. This detection limit was similar or lower than that of recently reported fiber probes [51,52,53]. There were no obvious Raman peaks found with 10^−3^ M CV using glass fiber directly (Figure 7a). The relationship between CV molecule concentrations and Raman intensities at 1619 cm^−1^ was linear (Figure 7b), which indicated promising potential quantitative analysis using these fiber probes. The Raman spectra of R6G showed six main peaks (Figure 7c) centered at 1651, 1509, 1361, 1310,1187, and 774 cm^−1^ which could be assigned to ring C-C stretching, N-H bending vibration, and out-of-plane vibration of ring C-H, respectively, from R6G molecules [54]. The 10^−8^ M lowest detectable limit and the linear R6G concentrations and Raman intensities at 1361 cm^−1^ (Figure 7d) indicated highly sensitive fiber probes for both qualitative and quantitative molecules detection.

The enhancement factor (EF) of fiber probe could be calculated using Equation (1) from Gupta [55].

EF = (I_SERS_/N_SERS_)/(I_neat_/N_neat_) (1)

Here, I_SERS_ and I_neat_ represent Raman signal intensities from SERS fiber probe and silica fiber, and N_SERS_, and N_neat_ are the molecules numbers involved in Raman signal generation from SERS fiber probe and silica fiber. The Raman intensities could be obtained from Raman spectra of 1.0 × 10^−8^ M CV using an SERS fiber probe and 0.1 M CV using silica fiber (Figure S6). The I _SERS_ and I neat were evaluated following the detailed processes provided by Liu et al. [56], with single-layer molecules assumption. The calculated EF was 6.23 × 10^6^, using CV analyte. Detailed calculation process is provided in the Supporting Information section.

**Figure 8:**
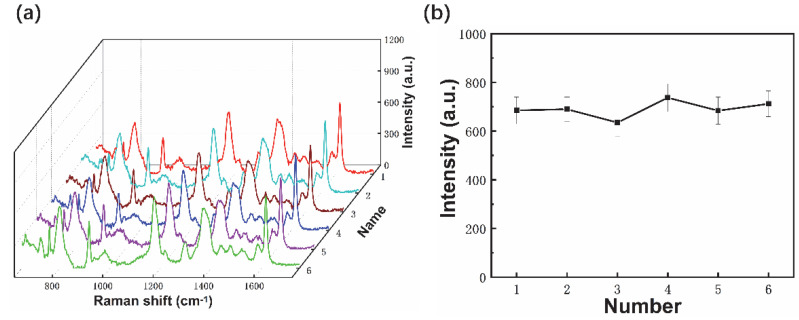
Raman spectra of 10^−5^ CV molecules (**a**) and 1619 cm^−1^ peak intensities (**b**) using same batch fiber probes.

The reproducibility of SERS fiber probes was characterized by the comparison of the intensities of Raman peaks from the same batch fiber probes fabricated with identical parameters using 10^−5^ M CV as an analyte. All of the Raman peaks could be assigned to different vibrations from CV and showed similar intensities (Figure 8a). The intensities of 1619 cm^−1^ peaks were around 690, albeit with a small difference. The related standard deviation was 4.93% for 10^−5^ CV, which indicated high reproducibility of the fabricated fiber probes and potential large-scale analysis applications.

**Figure 9:**
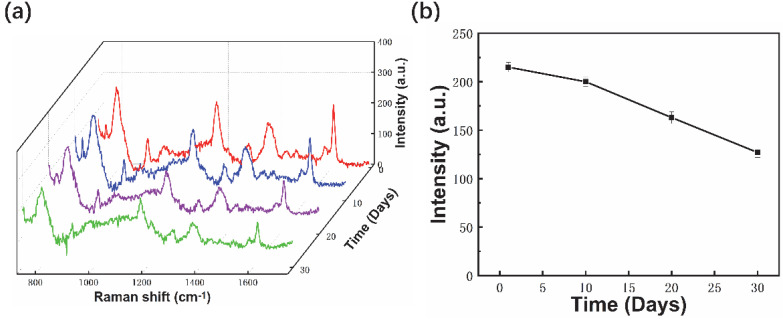
Raman spectra of 10^−6^ M CV molecules (**a**) and 1619 cm^−1^ peak intensities (**b**) using fiber probe exposed in air for different durations.

The stability of the fiber probes was characterized by exposed fiber probes in the air for 30 days at 25 °C and measured Raman spectra using 10^−6^ M CV every 10 days with the same parameters. The Raman peak intensities decreased with extended exposure durations (Figure 9a). The 1619 cm^−1^ peak intensities decreased from 210 to 160, or around 20% (Figure 9b). The fiber probes showed better stability than other silver nanostructure-based probes [57] for both the Au component and the special structure.

## 4. Conclusions

In conclusion, long spiky Au-Ag alloy nanostar-based fiber probes were fabricated, and SERS performances of the fiber probes were investigated in this paper. The nanostars were synthesized using the metal ions reduction method. The geometries, core sizes, and brunch tip length were controlled by LSB concentrations (as the nanostructure growth skeleton). The Au, and Ag element distributions in long spiky nanostars were inhomogeneous and there was a higher Ag content at the brunch tip top. The fiber probes were fabricated by fiber silanization and electrostatic adsorption. The detection limit of the fiber probes could reach 10^−8^ M for both CV and R6G molecules. The enhancement factor could be 6.23 × 10^6^ using CV as analyte. The relationship between analyte concentrations and Raman intensities was linear, which is beneficial for potential quantitative analysis. Moreover, the 4.93% related standard deviation for the same batch fiber probes indicated good reproducibility, and 20% Raman signal decay for 30-day exposure in air showed good stability of the fiber probes. The long spiky Au-Ag alloy nanostar-based fiber probes could be applied in low concentration substance detection.

## Data Availability

Not applicable.

## Supplementary Materials

The following supporting information can be downloaded at: https://www.mdpi.com/article/10.3390/ma15041498/s1. Figure S1. The diagram of SERS fiber probe fabrication. Figure S2. The 3D model of individual Au-Ag nanostar (a), colliding tip top nanostars (b), and two intertwined nanostars (c), Figure S3. The diagram of Raman measurement setup for SERS optical fiber probes, Figure S4. EDS spectrum of synthesized Au-Ag nanostars. The carbon, copper, and molybdenum were from copper mesh, carbon film and molybdenum ring for measurement. Table S1. The concentration of Au and Ag at different parts of nanostar in Figure 3a, Figure S5. The digital image of side view of silica fiber (a) and SEM image of fiber terminal facet (b), Figure S6. Raman spectrum of 10-8 M CV measured with SERS probe of long spiky Au-Ag alloy nanostars (a), Raman spectrum of 1.0 M CV measured with common multimode fiber (b). The peak centered at 1619 cm-1 was selected for EF calculation.
