# Supplementary material for: Long Spiky Au-Ag Nanostar Based Fiber Probe for Surface Enhanced Raman Spectroscopy
Source: Materials (Basel). 2022 Feb 17;15(4):1498. doi: 10.3390/ma15041498 (PMC8876936; doi:10.3390/ma15041498)
Supplement: Supplementary file 1 [file materials-15-01498-s001.zip › materials-1581389-supplementary.pdf]

materials

MDPI

Supplementary Materials

Long Spiky Au-Ag Nanostar Based Fiber Probe for Surface Enhanced Raman Spectroscopy

Guangyuan He [1], Xiaoyu Han [1], Shiyi Cao [1,2], Kaimin Cui [1,3], Qihang Tian [1] and Jihong Zhang [1,*]

[1] State Key Laboratory of Silicate Materials for Architectures, Wuhan University of Technology, 122 Luoshi Road, Wuhan 430070, China; heguangyuan@whut.edu.cn (G.H.); hxy0613@whut.edu.cn (X.H.); siyi@whut.edu.cn (S.C.); kaimin@whut.edu.cn (K.C.); tqh@whut.edu.cn (Q.T.)
[2] International School of Materials Science and Engineering, Wuhan University of Technology, 122 Luoshi Road, Wuhan 430070, China
[3] School of Materials Science and Engineering, Wuhan University of Technology, 122 Luoshi Road, Wuhan 430070, China
* Correspondence: optinfo@whut.edu.cn; Tel.: +86-27-8766-9729; Fax: +86-27-8766-9729

Citation: He, G.; Han, X.; Cao, S.; Cui, K.; Tian, Q.; Zhang, J. Long Spiky Au-Ag Nanostar Based Fiber Probe for Surface Enhanced Raman Spectroscopy. *Materials* **2022**, *15*, 1498. https://doi.org/10.3390/ma15041498

Academic Editor: Toma Stoica

Received: 18 January 2022
Accepted: 7 February 2022
Published: 17 February 2022

Publisher's Note: MDPI stays neutral with regard to jurisdictional claims in published maps and institutional affiliations.

Copyright: © 2022 by the authors. Licensee MDPI, Basel, Switzerland. This article is an open access article distributed under the terms and conditions of the Creative Commons Attribution (CC BY) license (http://creativecommons.org/licenses/by/4.0/).

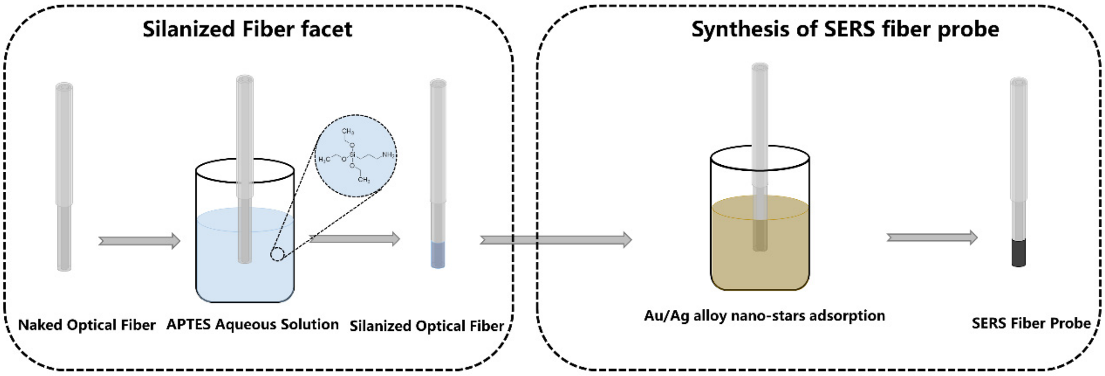

Figure S1. The diagram of SERS fiber probe fabrication.

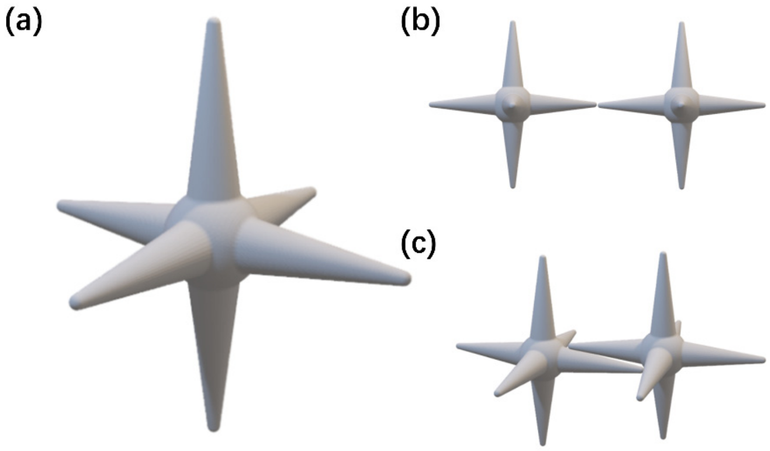

Figure S2. The 3D model of individual Au-Ag nanostar (**a**), colliding tip top nanostars (**b**), and two intertwined nanostars (**c**).

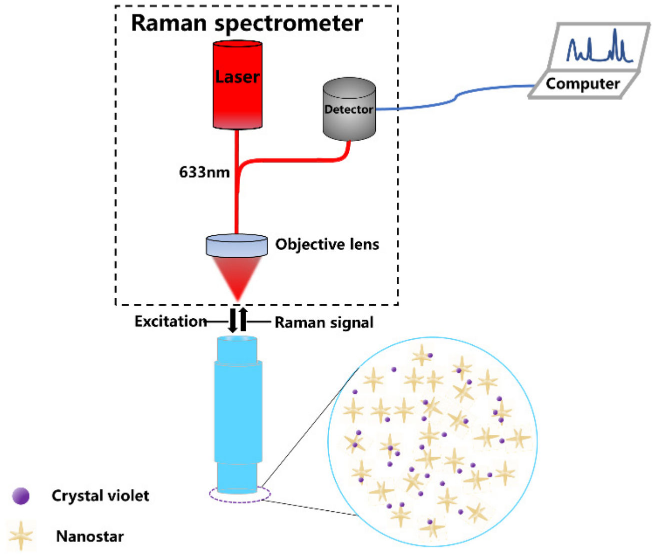

Figure S3. The diagram of Raman measurement setup for SERS optical fiber probes.

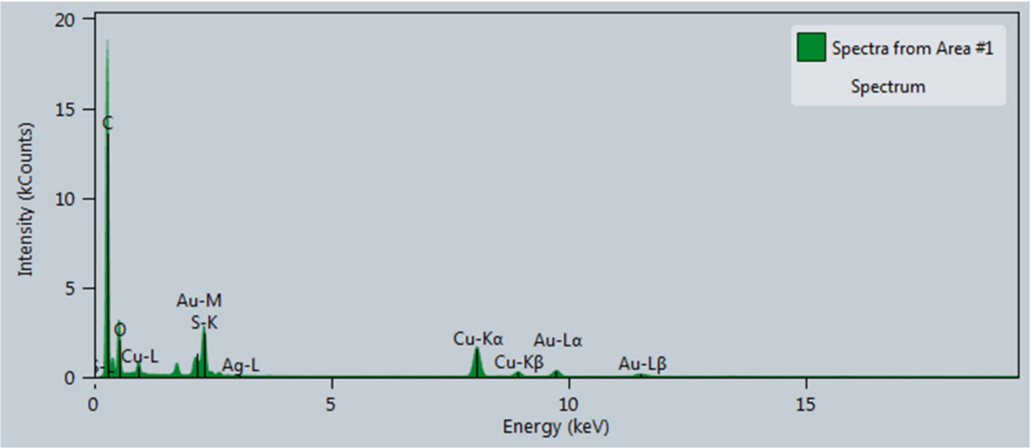

Figure S4. EDS spectrum of synthesized Au-Ag nanostars. The carbon, copper, and molybdenum were from copper mesh, carbon film and molybdenum ring for measurement.

Table S1. The concentration of Au and Ag at different parts of nanostar in Figure 3a.

Name	Element	Family	Atomic Fraction (%)	Mass Fraction (%)
Area 1	Ag	L	8.86	5.06
	Au	L	91.14	94.94
Area 2	Ag	L	8.75	4.99
	Au	L	91.25	95.01
Area 3	Ag	L	16.68	9.88
	Au	L	83.32	90.12
Area 4	Ag	L	17.92	10.68
	Au	L	82.08	89.32
Area 5	Ag	L	15.36	9.04
	Au	L	84.64	90.96
Area 6	Ag	L	16.56	9.80
	Au	L	86.44	90.20

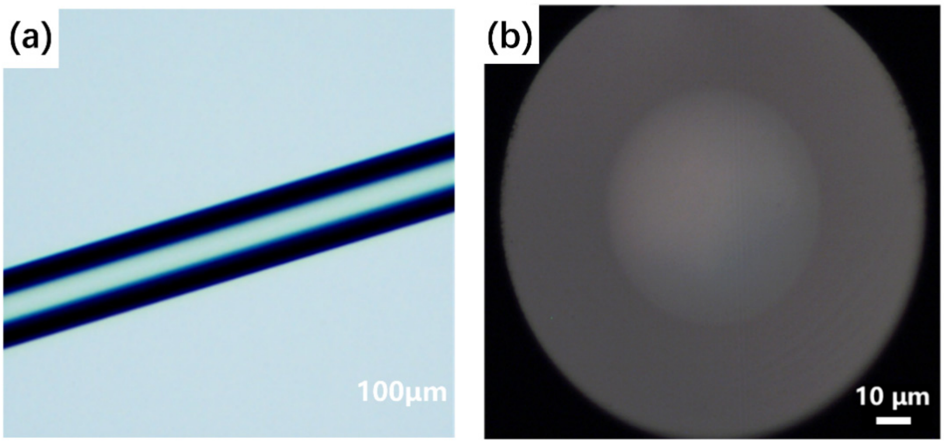

Figure S5. The digital image of side view of silica fiber (**a**) and SEM image of fiber terminal facet (**b**).

EF calculation:

The enhancement factor can be calculated by the equation:

$$EF = (I_{SERS} / N_{SERS}) / (I_{Raman} / N_{Raman})$$

Here, I_{SERS} and I_{Raman} represent Raman signal intensities from SERS fiber probe and silica fiber, N_{SERS}, and N_{Raman} are the molecules numbers involved in Raman signal generation from SERS fiber probe and silica fiber.

The crystal violet adsorbed on the surface of the metal substrate during characterization is assumed as monolayer film, so the above formula can be converted as follows:

$$EF = \frac{I_{SERS}}{A \times \frac{M_{SERS}}{S_{SERS}}} \times \frac{A \times \frac{M_{Raman}}{S_{Raman}}}{I_{Raman}} = \frac{I_{SERS} \times S_{SERS} \times M_{Raman}}{I_{Raman} \times S_{Raman} \times M_{SERS}}$$

In the formula, A represents the area of laser spot. Since both Raman characterization and SERS characterization tests were conducted under the same incident Angle of excitation wavelength and power, it can be considered that the same area of laser spot of Raman and SERS characterization tests. M_{Raman} and M_{SERS} are the number of analyte molecules adsorbed on the metal substrate of SERS probe and the surface of common fiber probe respectively. S_{SERS} and S_{Raman} are the geometric areas of analytes adsorbed on the surface

of fiber active probe and ordinary fiber probe respectively. S_{SERS} and S_{Raman} have the same values due to the consistent use of fiber material structure. The final analysis enhancement factor can be simplified into the following formula:

$$EF = \frac{I_{SERS} \times C_{Raman}}{I_{Raman} \times C_{SERS}}$$

where, C_{Raman} and C_{SERS} are analyte concentrations in conventional Raman and surface-enhanced Raman tests respectively. The conventional Raman test is to analyze the CV with a concentration of 0.1M by bare optical fiber, and the intensity of the characteristic peak at 1619 cm^{-1} is taken as the value of I. It worth to note that the enhancement factor calculation error of the is mainly from the monolayer assumption and I_{SERS} and I_{Raman} test error. The test error was minimized from the average value over multiple tests.

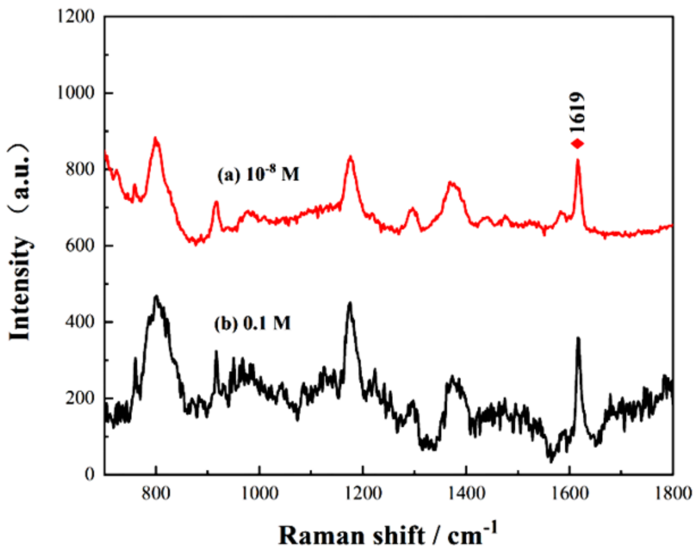

Figure S6. Raman spectrum of 10-8 M CV measured with SERS probe of long spiky Au-Ag alloy nanostars (**a**), Raman spectrum of 1.0 M CV measured with common multimode fiber (**b**). The peak centered at 1619 cm-1 was selected for EF calculation.
